# Significance of Pseudomeningocele After Decompressive Surgery for Chiari I Malformation

**DOI:** 10.3389/fsurg.2022.895444

**Published:** 2022-05-19

**Authors:** Artur Balasa, Przemysław Kunert, Mateusz Bielecki, Sławomir Kujawski, Andrzej Marchel

**Affiliations:** ^1^ Department of Neurosurgery, Medical University of Warsaw, Warsaw, Poland; ^2^ Department of Exercise Physiology and Functional Anatomy, Ludwik Rydygier Collegium Medicum in Bydgoszcz Nicolaus Copernicus University in Torun´, Bydgoszcz, Poland

**Keywords:** Chiari I malformation, pseudomeningocele, surgical and radiological outcomes, decompressive surgery, complications

## Abstract

**Background:**

Pseudomeningoceles (PMCs) as abnormal collections of cerebrospinal fluid are quite common findings on follow-up MRI after Chiari decompression surgery (CDS). However, the importance of their identification has not been truly determined, especially when PMCs are described occasionally in the process of radiological follow-up. We retrospectively analyzed surgical outcomes and imaging findings after CDS depending upon the occurrence and thickness of PMCs.

**Methods:**

A total of 76 adult patients who underwent CDS were analyzed. The clinical and radiological outcomes of patients with a pseudomeningocele (wPMC) were evaluated and compared to those of patients without a pseudomeningocele (w/oPMC). Radiological morphometric measurements were performed and compared between groups. Comparisons of the maximal PMC thickness were made within the wPMC group.

**Results:**

PMCs were recognized in 27 (35.5%) patients, of whom 3 (11.1%) required reoperation. Differences in satisfactory result rates regarding gestalt assessment and Chicago Chiari Outcome Scale were statistically insignificant between the w/oPMC and wPMC groups (*p* = 1 and *p* = 0.56, respectively). The postoperative syringomyelia decrease and cerebellar tonsil elevation were similar between the groups (*p* = 1 and *p* = 0.74, respectively) in the long-term follow-up. Additionally, the clinical or radiological outcomes with radiological details were not related to PMC thickness in the long-term follow-up. However, radiological details showed the cooccurrence of PMCs with a postsurgical of cerebello-tentorial distance increase (*p* < 0.05), basion-pontomedullary sulcus distance decrease (*p* < 0.05) and tonsillo-graft distance decrease (*p* < 0.05).

**Conclusions:**

We found no significant relationships between PMC presence or thickness and clinical or radiological outcomes. However, postoperative changes within the posterior fossa associated with PMCs resemble brain sagging, which occurs in intracranial hypotension. Therefore, extradural cerebrospinal fluid escape may also be responsible for symptoms in some patients with PMCs after CDS.

## Introduction

The Chiari malformations originally described by Hans Chiari in 1891 (1) relate to a rare group of hindbrain abnormalities concerning both pediatric and adult patients. The most common, Chiari I malformation (CMI), consists of caudal herniation of elongated cerebellar tonsils through the foramen magnum, causing symptoms secondary to compression of the brain stem, dysfunction of the cerebellum and distortion of cerebrospinal fluid (CSF) flow (2). Disturbances of CSF flow are responsible for syringomyelia, which has been reported in 69% of adult patients (3), but the exact pathophysiology remains unclear (4, 5). For symptomatic cases, the treatment of choice is suboccipital decompression with duraplasty (6). However, duraplasty is related to a larger number of complications with a lower recurrence rate of symptoms than osseous decompression alone (7–9). Average complication rates have been estimated at 4.5%, and among the most common causes of CSF leaks, aseptic meningitis and pseudomeningocele have been reported (3). Pseudomeningoceles (PMCs) are defined as abnormal CSF collections visible on MRI due to leakage into the extradural space (10) and directly over the dural graft. Pseudomeningocele can reduce the volume of reconstituted cisterna magna because of compression on duraplasty. Eventually, they can become symptomatic due to compression of neural structures or the impeding of CSF flow through the foramen magnum. Nevertheless, pseudomeningocele is not a rare finding on follow-up MRI, but it is not considered a complication until it leads to recurrent symptoms or CSF fistula or causes unacceptable cosmetic effects (11).

The aim of this study was to determine the roles of co-occurring pseudomeningoceles and their sizes on long-term outcomes after decompressive surgery with duraplasty in patients with CMI.

## Materials and Methods

A total of 96 adult patients who underwent posterior fossa decompression (PFD) with duraplasty for symptomatic CMI from January 2003 to December 2019 at our institution were screened. Twenty of them were excluded because their preoperative radiographic studies were not accessible. The mean Chicago Chiari Outcome Scale (CCOS) of the excluded and included patients did not significantly differ (12.85 vs. 12.4; *p* = 0.45). Of 76 included patients, 60 were women, and 16 were men, with an average age of 41.8 years old (range from 18 to 66 years old). The medical data were obtained from telephone questionnaires and hospital and ambulatory charts. For analysis of the long-term clinical course, gestalt assessment (improvement, unchanged or deterioration) and Chicago Chiari Outcome Scale (CCOS) were used (12–14). The mean clinical follow-up was 58 months.

All methods were carried out in accordance with relevant guidelines and regulations.

All protocols were approved by Bioethics Committee of Medical University of Warsaw (AKBE/231/2021). Informed consent was obtained from all subjects.

## Imaging

Preoperative and follow-up MRI was performed in every case. Follow-up MRI was scheduled 6 months after surgery, and additional studies were performed earlier or later, depending on the clinical indications. Numerous patients underwent many control MRI studies, especially those with long-term follow-up. In these cases, the last study was considered. The mean neuroimaging follow-up was 39.9 months.

The presence of pseudomeningocele on follow-up MRI was defined as hyperintense fluid collection above a hypointense linear dural graft on sagittal T2-weighted MRI imaging.

**Figure 1:**
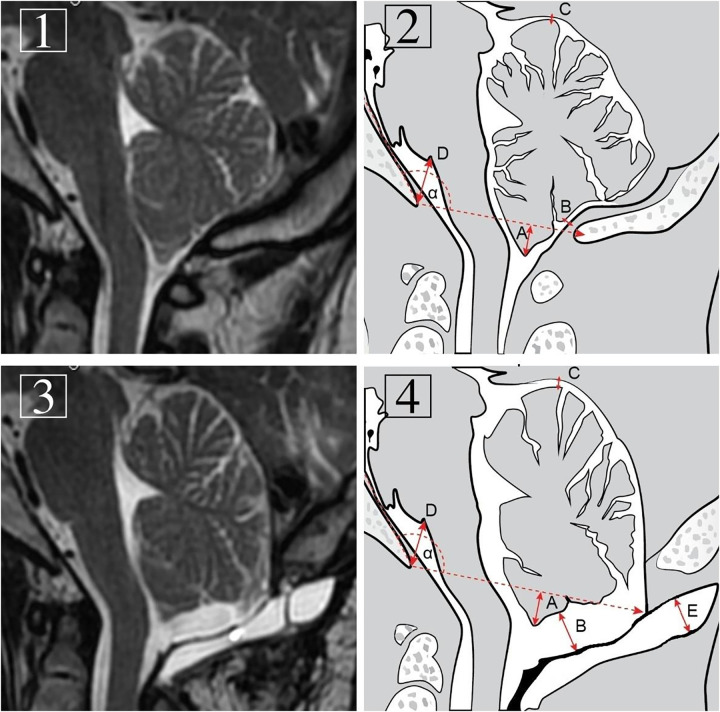
Preoperative (**1** and **2**) and follow-up (**3** and **4**) T1-weighted MRI images of the craniocervical junction region and diagrams presenting measurements in the same midsagittal plane. Measurements included: α: Boogaard's angle. A: max. tonsillar herniation. B: max. tonsillo-graft distance. C: max. cerebello-tentorial distance. D: max. basion-pontomedullary sulcus distance. E: pseudomeningocele thickness.

Because of the variable shape, precise measurement of PMC total volume is very difficult or impossible. Therefore, size was determined by the maximal thickness of the pseudomeningocele as the perpendicular distance to the graft with the best correlation clinically with complications (15) (**Figure 1**).

Maximal tonsillo-graft distance was also measured. Additional radiological details, such as pre- and postprocedural differences in maximal cerebello-tentorial distance and basion-pontomedullary sulcus distance, were noted. The levels of pre- and postoperative tonsillar descent were also measured and compared. For a postoperative tonsil position assessment, the level of the foramen magnum was determined by restoration of the angle between the line tangent to the clivus surface and the basion-opisthion line established on preoperative images (Boogaard’s angle; **Figure 1**) (16). In cases of syringomyelia in the preoperative study, their size was determined to be decreased, stable or increased. The cooccurrence of pseudomeningocele and its maximal thickness and clinical and radiological outcomes were analyzed in detail, including the poor preoperative status of 3 reoperated patients for symptomatic PMCs. Evaluation of the relationship of PMC thickness with tonsillo-graft distance and pre- and postprocedural differences in maximal cerebello-tentorial distance, basion-pontomedullary sulcus distance and tonsillar herniation was performed. In cases of pre- and postoperative distance comparisons, we received positive or negative values depending on whether a particular distance decreased or increased on follow-up MRI, respectively (**Figure 1**).

## Surgical Technique

Suboccipital craniectomy with C1 posterior arch removal and sometimes with partial (No. 24) or whole (No. 6) C2 laminectomy was performed with Y-shaped dural incision and with subsequent duraplasty in all cases. Depending on surgeon preference, two types of graft material were used for dural closure: nonautologous grafts or autologous grafts. The former, represented by synthetic collagen matrices, was used in 48 (63.2%) cases. Autologous grafts in this series, including previously harvested pericranium or fascia lata, were used in 28 (36.8%) cases. The arachnoid layer, 4^th^ ventricle and tonsils were left intact, and no local or lumbar drains after surgery were used (17, 18).

## Statistical Analysis

The Shapiro-Wilk test was used to test the assumption of normality, and Levene’s test was used to examine the assumption of homogeneity. Mean values and standard deviations (SDs) are reported. Fisher's exact test was used to examine the relationship between PMC presence and changes in symptom severity expressed in a binary manner, while the chi-square test was used in the case of symptom severity changes in three categories (improvement vs. unchanged vs. deterioration). The Mann-Whitney U test was used to compare values of continuous and ordinal variables between independent groups. The significance level was set at alpha = 0.05.

## Results

Twenty-seven (35.5%) patients demonstrated PMC on posttreatment MRI. Only 3 (11.1%) of 27 patients required revision surgery, representing 3.9% of the overall study cohort. Satisfactory clinical results according to gestalt assessment and CCOS were obtained in 75.5% and 81.6% of patients without pseudomeningocele (w/oPMC) and 66.7% and 70.4% of patients with pseudomeningocele (wPMC), respectively (*p* = 0.43, *p* = 0.27). For this outcome analysis, the prereoperative clinical condition of 3 reoperated patients was considered to capture the worst condition potentially related to PMC. Long-term follow-up showed even more comparable satisfactory results: 75.5% vs. 77.7% (*p* = 1) in gestalt and 81.6% vs. 77.7% (*p* = 0.56) in CCOS score for w/oPMC and wPMC patients, respectively (**Table 1**). Analysis of individual signs and symptoms showed no significant correlations between the wPMC and w/oPMC groups postoperatively (Supplementary Table S1).

**Table 1:** Comparison of clinical and radiological outcomes between patients without pseudomeningocele (w/oPMC) and with pseudomeningocele (wPMC) after decompression surgery in patients with Chiari I malformation, including preoperative status of 3 patients reoperated due to PMC and over long-term follow-up.

Comparison clinical and radiological outcomes, including preoperative status of 3 patients reoperated due to PMC				
Surgical outcomes		Patients w/oPMC 49 pts. number of pts. (%)	Patients wPMC 27 pts. number of pts. (%)	*p-*value
Gestalt	Improvement or unchanged	37 (75.5%)	18 (66.7%)	0.43
	Deterioration	12 (24.5%)	9 (33.3%)	
CCOS^a^	≥12 (satisfactory)	40 (81.6%)	19 (70.4%)	0.27
	<12 (unsatisfactory)	9 (18.4%)	8 (29.6%)	
Syringomyelia on follow-up MRI (56 pts.)		Patients w/oPMC with syringomyelia 36 pts. number of pts. (%)	Patients wPMC and syringomyelia 20 pts. number of pts. (%)	*p-*value
Improvement		33 (91.7%)	15 (75.0%)	0.12
Stable or deterioration		3 (8.3%)	5 (25.0%)	
Comparison of long- term clinical and radiological outcomes				
Long-term surgical outcomes		Patients w/oPMC 49 pts. number of pts. (%)	Patients wPMC 25 pts. number of pts. (%)	*p-*value
Gestalt	Improvement or unchanged	37 (75.5%)	19 (77.7%)	1
	Deterioration	12 (24.5%)	6 (22.3%)	
CCOS^a^	≥12 (satisfactory)	40 (81.6%)	19 (77.7%)	0.56
	<12 (unsatisfactory)	9 (18.4%)	6 (22.3%)	
Syringomyelia on follow-up MRI (56 pts.)		Patients w/oPMC with syringomyelia 37 pts. number of pts. (%)	Patients wPMC and syringomyelia 19 pts. number of pts. (%)	*p-*value
Improvement		33 (89.2%)	17 (89.5%)	1
Stable or deterioration		4 (10.8%)	2 (10.5%)	

^*a*^ *Chicago Chiari Outcome Scale.*

Syringomyelia was present in 73.7% of patients preoperatively. A reduction in syringomyelia size was obtained in 91.7% of cases in the w/oPMC group and 75.0% of cases in the wPMC groups (*p* = 0.12). Two patients who underwent reoperation due to PMC had syringomyelia that remained unchanged after the first operation. In the long-term follow-up, including the results of 3 revision surgeries, the decrease in syrinx was similar: 89.2% vs. 89.5% for the w/oPMC and wPMC groups, respectively (*p* = 1; **Table 2**).

**Table 2:** Comparison of long-term radiological details on follow-up MRI between patients without pseudomeningocele (w/oPMC) and with pseudomeningocele (wPMC) after decompression surgery in patients with Chiari I malformation.

Radiological details on follow-up MRI	Patients w/oPMC 49 pts. mean [mm] ± SD	Patients wPMC^a^ 27 pts. mean [mm] ± SD	*p-*value
Tonsillo-graft distance	7.1 ± 5.4	4.4 ± 3.3	<0.05
Pre- and postoperative difference of cerebello-tentorial distance	0.3 ± 1.0	−1.2 ± 1.4	<0.05
Pre- and postoperative difference of basion-pontomedullary sulcus distance	−0.6 ± 1.6	0.6 ± 1.5	<0.05
Pre- and postoperative difference of tonsillar herniation	4.1 ± 3.9	3.7 ± 3.1	0.74

^a^ *Including the patient’s clinical status before reoperation due to PMC.*

Pseudomeningocele was associated with a reduction in the average maximal tonsillo-graft distance on follow-up MRI (4.4 mm vs. 7.1 mm; *p* < 0.05). The cerebello-tentorial distance increased postsurgically in the wPMC group by an average of 1.2 mm, in contrast to the patients with w/oPMC, in whom it slightly decreased by an average of 0.3 mm (*p* < 0.05). Additionally, the existence of PMC was associated with a decrease in basion-pontomedullary sulcus distance by an average of 0.6 mm compared to the w/oPMC group, in which it was increased by an average of 0.6 mm (*p* < 0.05). However, we did not observe a substantial difference in tonsil elevation after surgery between the w/oPMC and wPMC groups on follow-up MRI (4.1 mm vs. 3.7 mm; *p* = 0.74; **Table 3**).

**Table 3:** Correlation between pseudomeningocele thickness on follow-up MRI related and clinical outcome in CCOS (Chicago Chiari Outcome Scale) and radiological details.

PMC^b^ thickness	2–7 mm	≥8 mm	*p-*value
No. of patients	13	14	
Mean CCOS^a^ ±SD	11.4 ± 3.5	12.6 ± 2.7	0.39
Pre- and postoperative difference of tonsillo-graft distance [mm] ± SD	5.1 ± 3.8	3.8 ± 2.7	0.31
Pre- and postoperative difference of cerebello-tentorial distance [mm] ± SD	−1.5 ± 1.5	−0.9 ± 1.5	0.18
Pre- and postoperative difference of basion-pontomedullary sulcus distance [mm] ± SD	0.4 ± 1.9	0.8 ± 1.4	0.52
Pre- and postoperative difference of tonsillar herniation [mm] ± SD	3.9 ± 2.8	3.5 ± 3.5	0.75
Syringomyelia on follow-up MRI (20 pt.)	PMC^b^ thickness		
	Patients with syringomyelia [9 pts.] No. of pts. (%)	Patients with syringomyelia [11 pts.] No. of pts. (%)	
Improvement	8 (88.9%)	8 (72.7%)	0.59
Stable or deterioration	1 (11.1%)	3 (27.3%)	

^a^ *Including the patient’s clinical status before reoperation due to PMC.*

^*b*^ *Pseudomeningocele.*

The average thickness of PMC was 8.7 mm ± 4.5 (SD) (range 2.0–21.0 mm). Relationships of PMC thickness with CCOS, individual signs and symptoms were not statistically significant (Supplementary Table S1). Additionally, we did not find an association between PMC thickness and changes in syrinx size on follow-up (*p* = 0.59) or other radiological details (**Table 3**).

A distinct group consisted of 3 reoperated patients due to symptomatic PMCs. Significant cerebellar subsidence coexisted in 2 patients. The average time between the operation and the onset of new symptoms was 7.7 days (range: 3–13). The predominant symptoms were severe headache, nausea, and vomiting with depressed levels of consciousness. Reduraplasty was performed in all cases, with optimization of craniectomy size in 2. The clinical condition assessed with the CCOS in these cases before reoperation was significantly worse than that of the rest of the wPMC group (7.3 vs. 12.3; *p* = 0.02; **Table 4**). The mean CCOS of the reoperated patients improved in the long-term follow-up to 13.7 (range: 12–15), although persistent PMC was noted in 1 case on follow-up MRI. Comparison of radiological details of the reoperated PMCs to the remaining PMCs in the PMC group showed an insignificantly larger mean PMC thickness (*p* = 0.37) and a smaller reduction in tonsillo-graft distance (*p* = 0.73) in the reoperated patients. Prepostoperative differences in tonsillar herniation (*p* = 0.27), cerebello-tentorial distance (*p* = 0.20), and basion-pontomedullary sulcus distance (*p* = 0.73) were insignificantly less favorable in the reoperated patients than in nonreoperated patients with PMC (**Table 4**). We have not observed external CSF leaks in our series.

**Table 4:** Comparison of clinical outcomes and radiological details on follow-up MRI between reoperated and nonreoperated patients for pseudomeningocele (wPMC) after decompression surgery in patients with Chiari I malformation.

	Patients with PMC^b^		*p-*value
	Reoperated due to PMC (No. 3)	Nonreoperated (No. 24)	
Mean CCOS^a^ [mm] ± SD	7.3 ± 1.2	12.3 ± 2.8	<0.05
PMC^b^ thickness [mm] ± SD	11.3 ± 5.9	8.3 ± 4.5	0.37
Pre- and postoperative difference tonsillo-graft distance [mm] ± SD	3.3 ± 1.5	4.5 ± 3.4	0.73
Pre- and postoperative difference cerebello-tentorial distance [mm] ± SD	−3.7 ± 0.5	−0.9 ± 1.3	0.20
Pre- and postoperative difference basion-pontomedullary sulcus distance [mm] ± SD	0.2 ± 0.5	0.6 ± 1.8	0.73
Pre- and postoperative difference tonsillar herniation [mm] ± SD	1.2 ± 3.3	4.0 ± 3.0	0.27

^a^ *Chicago Chiari Outcome Scale.*

^*b*^ *Pseudomeningocele.*

## Discussion

Surgical treatment for CMI has a particular predisposition to the development of CSF-related complications, including PMCs. Among predisposing factors worth mentioning are duraplasty, craniectomy, midline approach, and surgery concerning the posterior fossa (10, 15, 19). Thus, PMCs are among the most common complications after Chiari decompression surgery (20). Smith et al. noted that CM was the second most common cause of PMC after surgery for posterior fossa extraaxial tumors (10). PMC rates reported in the literature range from 2.5 to 24% (15, 21, 22). We observed PMCs in 35.5% of cases. However, we assessed even barely visible PMCs on follow-up MRI to define their exact significance. This wide disparity presumably resulted from some authors having considered PMCs recognized only on imaging studies as asymptomatic or incidental findings and not, therefore, reporting them as complications (10, 21).

According to the current state of knowledge, the appearance of PMC at the operation site might have no effect on duraplasty, or it can lead to slight reduction in recreated cisterna magna or obstruction of CSF flow, potentially leading to hydrocephalus (11). Further PMC enlargement can lead to compression of the posterior fossa neural structures. The patient either remains stable, or new symptoms can appear. PMC manifestation evolves from local pain in distended tissue or simple headache to posterior fossa syndrome or even impaired consciousness. Moreover, PMC can lead to CSF fistula and meningitis in cases of skin rupture (19).

The pathophysiology of PMC formation is unclear, but it seems that PMCs are initially formed as a result of suture CSF leakage between the dura and dural grafts or a tear in one of them. Leakage can occur immediately after an operation due to poor dural closure or could be caused by a progressive increase in intracranial CSF pressure in the course of hydrocephalus (23). However, knowing the role of CSF pressure, Valsalva maneuvers (e.g., coughing, sneezing, or defecating) likely cause a sudden increase in pressure, acting as a trigger factor in leakage (20). Symptomatic PMCs are most often observed shortly after surgery, which would suggest their onset until strong scarring among the graft, dura and neck muscles is created (15, 24).

Comparison of postoperative radiographic images of patients without and with PMC showed significant differences. A tendency to decrease the brainstem with the cerebellum in the wPMC group, defined as increased cerebello-tentorial and reduced basion-pontomedullary sulcus distances, was noted. This tendency was the opposite to that observed in the w/oPMC group, in which these structures had ascended (**Table 2**).

Considering our findings, we propose that the clinical consequences of some PMCs could develop via a similar mechanism to that of spontaneous intracranial hypotension (SIH). Spontaneous CSF fistula manifests as a small CSF reservoir (meningeal diverticula), usually associated with nerve root sleeves. CSF leakage is self-limiting, or CSF constantly leaks out in intracranial hypotension, e.g., after lumbar puncture (25–27). Similarly, postoperative leakage usually does not result in a constant increase in PMC volume. Similar to brain sagging in SIH, we noted slight hindbrain subsidence in the wPMC group, expressed as a postoperative increase in the supracerebellar space and a decrease in the basion-pontomedullary sulcus distance (**Figure 1**). The cooccurrence of cerebello-tentorial distance increases and basion-pontomedullary sulcus distance decreases in patients with PMCs might derive from the pressure gradient between the posterior fossa and PMC spaces, with a subsequent downward shift of all posterior fossa neural structures. Hypotension in the posterior fossa corresponds to partial hypotension syndrome but is not as severe as SIH in causing sagging of the whole brain (25, 28). This difference might indicate that postoperative cerebellar subsidence could be related not only to oversized occipital bony decompression but also to CSF leakage.

Raising and changing the shape of primarily herniated tonsils after decompression surgery from thin and extended to rounder and shorter are well known (16). Interestingly, despite sagging of the superior surface of the cerebellum in patients wPMC, we did not observe a significant difference in postsurgical tonsil tip ascent between the two groups, suggesting that favorable and unfavorable displacements in the posterior fossa might coexist after decompression.

Although the recreated cisterna magna was significantly smaller in patients with PMC, and the worst “prerevision” condition related to PMC was used for analysis, the comparison of the w/oPMC and wPMC groups showed no significant differences in clinical outcomes. Furthermore, the long-term observation of 25 patients with persistent PMC demonstrated that they achieved very similar clinical and radiological outcomes as their counterparts without PMC. It appears that the relative reduction in the recreated intradural space was not sufficient to block CSF flow in nonreoperated patients with PMC (29).

Pare and Batzdorf reported three cases in which PMCs were a reason for persistent syringomyelia in long-term follow-up (30). In our series, in which it was never a main cause of reoperation for PMC, all 3 revisions were performed too early after the first surgery to expect a decrease in the syrinx. We did not find any impact of the presence or size of PMCs on syringomyelia evolution.

Thus far, based on our analysis, neither the presence nor any size of PMC can be identified as a risk factor for worse outcomes. All PMCs requiring revision manifested shortly after surgery, long before scheduled follow-up MRI. Therefore, early clinical postsurgical deterioration is much more suggestive of PMC importance than any long-term radiological parameter. However, the differences in hindbrain rearrangement after decompression between patients with and without PMC shed new light on the potential mechanism increasing symptoms from some PMCs.

## Limitations

Our study is limited by several factors, the most significant of which is the retrospective nature and single-center design of the research. Moreover, the cohort was represented by only 76 patients, with a significant effect on the statistical analysis of certain differences between groups. Additionally, the radiological data were based on MRI studies performed at various times after surgery and sometimes with different resolution of studies, which might have had an impact on the measured details. Standard follow-up MRI was performed without contrast, which could have shown other intracranial hypotension features. However, we obtained a relatively long-term follow-up by choosing to measure the last available MRI study. Future prospective research with a larger cohort is needed, especially to confirm our observations contained in the conclusion.

## Conclusion

We did not find any significant relationships of pseudomeningocele presence or pseudomeningocele thickness with clinico-radiological outcomes after decompressive surgery. In rare cases, PMCs might be a cause of clinical deterioration over short postoperative periods. However, the symptoms could be secondary to hindbrain lowering caused by posterior fossa hypotension, resulting from extradural CSF leakage, rather than from narrowing of the intradural space at the foramen magnum level.

## Data Availability

The original contributions presented in the study are included in the article/Supplementary Material, further inquiries can be directed to the corresponding author/s.
