# Supplementary material for: Significance of Pseudomeningocele After Decompressive Surgery for Chiari I Malformation
Source: Front Surg. 2022 May 19;9:895444. doi: 10.3389/fsurg.2022.895444 (PMC9406808; doi:10.3389/fsurg.2022.895444)
Supplement: Supplementary file 3 [file Table_1_v1.docx]

|  | **w/oPMC** | | | **wPMC** | | | **p value** |
| --- | --- | --- | --- | --- | --- | --- | --- |
| **Preoperative**  **signs and symptoms** | Improvement | Unchanged | Deterioration | Improvement | Unchanged | Deterioration |  |
| **Suboccipital or general headache** | 22 (64.7%) | 6  (17.6%) | 6  (17.6%) | 15  (65.2%) | 4  (17.4%) | 4  (17.4%) | 1 |
| **Neck and/or shoulder and arm pain** | 8  (38.1%) | 5  (23.8%) | 8  (38.1%) | 12  (31.6%) | 11  (28.9%) | 15  (39.5%) | 0.86 |
| **Decreased temperature sensitivity** | 9  (37.5%) | 13  (54.2%) | 2  (8.3%) | 4  (40%) | 6  (60%) | 0  (0%) | 0.64 |
| **Decreased touch sensitivity** | 1  (6.7%) | 11  (73.3%) | 3  (20%) | 1  (8.3%) | 9  (75%) | 2  (16.7%) | 0.97 |
| **Decreased pain sensitivity** | 3  (16.7%) | 14  (77.8%) | 1  (5.5%) | 2  (22.2%) | 6  (66.7%) | 1  (11.1%) | 0.80 |
| **Somatosensory disturbances**  **(paresthesia, hyperesthesia)** | 8  (27.6%) | 13  (44.8%) | 8  (27.6%) | 4  (25%) | 10  (62.5%) | 2  (12.5%) | 0.42 |
| **Paresis or motor weakness** | 3  (12.5%) | 14  (58.3%) | 7  (29.2%) | 2  (15.4%) | 7  (53.8%) | 4  (30.8%) | 0.96 |
| **Cerebellar signs (nystagmus, ataxia, dizziness, dysarthria, imbalance)** | 10  (37.0%) | 9  (33.3%) | 8  (29.6%) | 7  (53.8%) | 2  (15.4%) | 4  (30.8%) | 0.45 |
| **Dysphagia** | 4  (44.4%) | 5  (55.6%) | 0  (0%) | 2  (18.2%) | 6  (54.5%) | 3  (27.3%) | 0.17 |

**Supplementary Table 1**. Long-term results for preoperative signs and symptoms in the without pseudomenigocele (w/oPMC) versus pseudomenigocele (wPMC) groups.
